# The ω‐3 fatty acid α‐linolenic acid extends *Caenorhabditis elegans* lifespan via NHR‐49/PPARα and oxidation to oxylipins

**DOI:** 10.1111/acel.12651

**Published:** 2017-08-03

**Authors:** Wenbo Qi, Gloria E. Gutierrez, Xiaoli Gao, Hong Dixon, Joe A. McDonough, Ann M. Marini, Alfred L. Fisher

**Affiliations:** ^1^ Center for Healthy Aging University of Texas Health Science Center at San Antonio San Antonio TX 78229 USA; ^2^ Pharmaceuticals and Bioengineering Chemistry and Chemical Engineering Division Southwest Research Institute San Antonio TX 78238 USA; ^3^ Department of Biochemistry University of Texas Health Science Center at San Antonio San Antonio TX 78229 USA; ^4^ Department of Neurology and Program in Neuroscience Uniformed Services University of the Health Sciences Bethesda MD 20814 USA; ^5^ Division of Geriatrics, Gerontology, and Palliative Medicine Department of Medicine University of Texas Health Science Center at San Antonio San Antonio TX 78229 USA; ^6^ GRECC South Texas VA Healthcare System San Antonio TX 78229 USA

**Keywords:** aging, *Caenorhabditis elegans*, SKN‐1, NHR‐49, ω‐3 fatty acids, oxylipin

## Abstract

The dietary intake of ω‐3 polyunsaturated fatty acids has been linked to a reduction in the incidence of aging‐associated disease including cardiovascular disease and stroke. Additionally, long‐lived *Caenorhabditis elegans glp‐1* germ line‐less mutant animals show a number of changes in lipid metabolism including the increased production of the ω‐3 fatty acid, α‐linolenic acid (ALA). Here, we show that the treatment of *C. elegans* with ALA produces a dose‐dependent increase in lifespan. The increased longevity of the *glp‐1* mutant animals is known to be dependent on both the NHR‐49/PPARα and SKN‐1/Nrf2 transcription factors, although the mechanisms involved are incompletely understood. We find that ALA treatment increased the lifespan of wild‐type worms and that these effects required both of these transcription factors. Specifically, NHR‐49 was activated by ALA to promote the expression of genes involved in the β‐oxidation of lipids, whereas SKN‐1 is not directly activated by ALA, but instead, the exposure of ALA to air results in the oxidation of ALA to a group of compounds termed oxylipins. At least one of the oxylipins activates SKN‐1 and enhances the increased longevity resulting from ALA treatment. The results show that ω‐3 fatty acids inhibit aging and that these effects could reflect the combined effects of the ω‐3 fatty acid and the oxylipin metabolites. The benefits of ω‐3 fatty acid consumption on human health may similarly involve the production of oxylipins, and differences in oxylipin conversion could account for at least part of the variability found between observational vs. interventional clinical trials.

## Introduction

The ω‐3 fatty acids are characterized by desaturation at the third carbon from the methyl, or ω‐carbon, end of the molecule, and the most biologically relevant ω‐3 fatty acids include α‐linolenic acid (ALA), eicosapentaenoic acid (EPA), and docosahexaenoic acid (DHA) (reviewed in von Schacky, 1987). In people, the ω‐3 fatty acids are essential, and common dietary sources include plants for ALA and fish or fish oils for EPA and DHA. Interest in these polyunsaturated fatty acids increased greatly after epidemiologic studies linked greater fish intake to a greatly reduced rate of hypertension and atherosclerotic cardiovascular disease (von Schacky, 1987). Subsequent basic and translational science studies suggested that these benefits could be due to changes in eicosanoid production favoring reductions in vascular reactivity and blood clotting along with favorable changes in serum cholesterol levels. However, these benefits have been difficult to reproduce in clinical trials using dietary ω‐3 fatty acid supplements, and more recent analyses of the dietary studies have suggested that the benefits of dietary ω‐3 fatty acid intake may be more modest than previously reported (Chowdhury *et al*., 2014). Nevertheless, it is possible that the augmentation of ω‐3 fatty acid intake could produce multiple health benefits, particularly if the optimal dose and dosing regimen can be rigorously established and if those individuals that are most likely to benefit could be more readily be identified (Weylandt *et al*., 2015).

In *C. elegans,* the loss of germ line stem cells produces an increase in lifespan that depends on a number of downstream changes in the animals including the activation of the *daf‐16*/FOXO transcription factor, the *nhr‐49*/PPARα nuclear hormone receptor, the *nhr‐80/*HNF4 nuclear hormone receptor, the *daf‐12* nuclear hormone receptor, and the *skn‐1*/Nrf2 transcription factor (Hsin & Kenyon, 1999; Goudeau *et al*., 2011; Ratnappan *et al*., 2014; Steinbaugh *et al*., 2015). One of the effects of the loss of germ line stem cells in *glp‐1* mutants is the augmentation of both lipid synthesis and lipid metabolism via β‐oxidation, which produces a net increase in fat content (O'Rourke *et al*., 2009; Ratnappan *et al*., 2014; Steinbaugh *et al*., 2015; Amrit *et al*., 2016). In particular, a number of lipids show *a daf‐16‐* and/or *nhr‐49*‐dependent increase in synthesis in the *glp‐1* mutant animals including oleic acid, linoleic acid, and the ω‐3 fatty acid linolenic acid (Ratnappan *et al*., 2014; Amrit *et al*., 2016). Further, the degradation of fatty acids via β‐oxidation is required for the increased longevity of the *glp‐1* mutant animals (Ratnappan *et al*., 2014). Hence, an important aspect of the response to the loss of germ line stem cells is a metabolic reprogramming with the enhanced production of specific lipids and a shift toward respiration fueled by β‐oxidation.

Interestingly, products of lipid metabolism can also act independently to promote longevity in the *glp‐1* mutant worms. For example, the production of the ω‐9 fatty acid oleic acid, promotes longevity, at least in part, through *skn‐1* activation, which suggests that the changes in lipid metabolism can play a role in either activating or reinforcing the activity of these transcription factors (Goudeau *et al*., 2011; Steinbaugh *et al*., 2015). Additionally, the lysosomal lipase, *lipl‐4*, is induced in the *glp‐1* mutant animals and promotes longevity via the release of a number of lipids including the ω‐6 fatty acids arachidonic acid or di‐homo‐γ‐linoleic acid (Wang *et al*., 2008; O'Rourke *et al*., 2013; Folick *et al*., 2015). These lipids have multiple effects in worms including the activation of autophagy and also promoting the transcriptional activity of the *nhr‐49* and *nhr‐80* transcription factors (Lapierre *et al*., 2011; O'Rourke *et al*., 2013; Folick *et al*., 2015). Consequently, study of the germ line mutants in *C. elegans* has uncovered new effects of lipids on the aging process and identified distinct downstream pathways activated by each.

Here, we test whether the treatment of worms with the ω‐3 fatty acid α‐linolenic acid (ALA) affects the lifespan of *C. elegans*. We find that ALA treatment increases the lifespan of treated worms and that these effects occur via a differing mechanism than those involved in the effects of ω‐6 fatty acids and involve the activation of both the *nhr‐49* nuclear hormone receptor and the *skn‐1* transcription factor. Further, we find that the ability of ALA treatment to activate *skn‐1* may depend on the level of spontaneous oxidation of ALA to a number of oxylipin derivatives. Together, our results demonstrate a novel effect of ω‐3 fatty acids on the aging process and emphasize the important changes that fatty acids can undergo outside of the classic synthetic and degradation pathways.

## Results

### α‐linolenic acid treatment increases *C. elegans* lifespan

**Figure 1:**
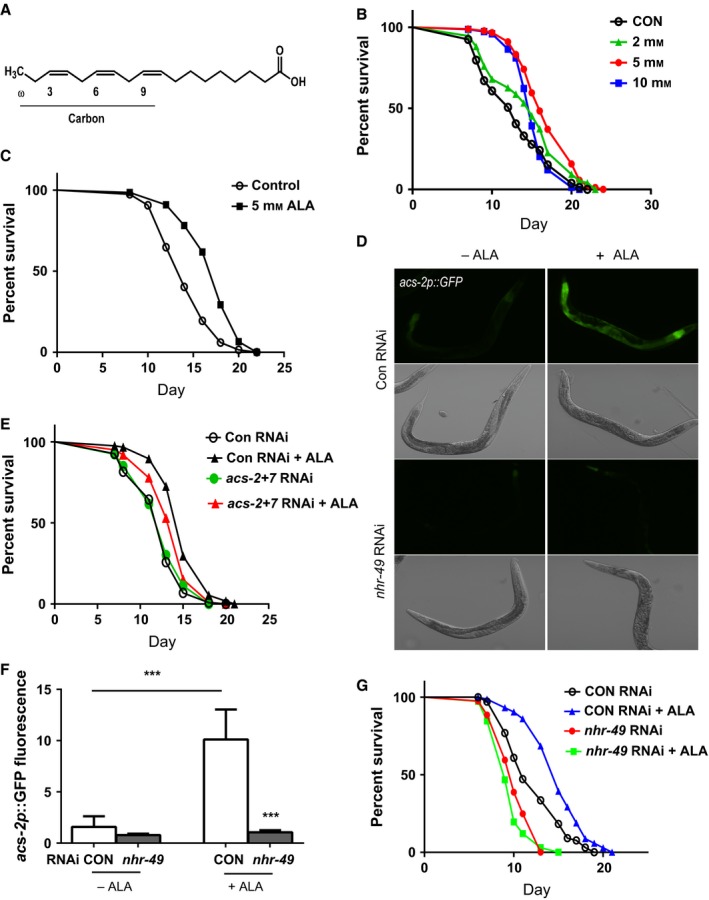
α‐linolenic acid (ALA) treatment increases worm lifespan via *nhr‐49*/PPARα. (A) Structure of ALA showing the locations of the desaturated carbon bonds in the fatty acid backbone. (B) Treatment of *spe‐9; fer‐15* worms with ALA at concentrations of 2–10 mm increases lifespan, compared to control animals treated with the ALA solvent alone, with the maximal effect being observed at the 5 mm dose. *N* > 80 for all treatments. *P* < 0.0001 for control vs. 5 mm ALA treatment by log‐rank test. (C) Treatment of wild‐type N2 worm with 5 mm ALA increases lifespan, compared to control animals treated with the ALA solvent alone. *N* > 75 for both treatments. *P* < 0.0001 by log‐rank test. The expression of an *acs‐2p::GFP* reporter is increased after treatment of 5 mm ALA as shown by fluorescence microscopy (D) or quantitation of the GFP fluorescence in the images using the ImageJ program (F). The increase in expression requires *nhr‐49*/PPARα as the increase in expression is blocked by *nhr‐49* RNAi. *N* > 6 for all RNAi and ALA treatment combinations. *** represents *P* < 0.001 for Control RNAi ‐ ALA vs. Control RNAi +ALA and Control RNAi +ALA vs. *nhr‐49* RNAi + ALA by *t*‐test. (E) Inhibition of *acs‐2* and *acs‐7* in *spe‐9; fer‐15* worms by RNAi reduces the effects of ALA treatment on lifespan. *N* > 100 for all treatments. *P* = 0.0006 for *acs‐2* + *acs‐7* RNAi ALA treated vs. control RNAi ALA treated by log‐rank test. *P* = NS for *acs‐2* + *acs‐7* RNAi control treated vs. control RNAi control treated by log‐rank test. *P* < 0.0001 for control RNAi control treated vs. control RNAi ALA treated. (G) The increase in worm lifespan produced by treatment with 5 mm ALA requires *nhr‐49* because *spe‐9; fer‐15* worms treated with *nhr‐49* RNAi show no increase in lifespan following ALA treatment. *N* > 72 for all treatments. *P* < 0.0001 for control RNAi −ALA vs. +ALA and *P* = NS for *nhr‐49* RNAi −ALA vs. +ALA.

The ω‐3 fatty acid α‐linolenic acid (ALA) is a polyunsaturated fatty acid that consists of an 18 carbon chain with *cis* unsaturated double bonds at the ω‐3, ω‐6, and ω‐9 positions where the ω carbon is at the opposite end of the lipid from the carboxyl group (Fig. 1A). To determine the effects of ALA treatment on lifespan, we treated worms with a range of ALA concentrations ranging from 2 to 10 mm. We found that all concentrations significantly increased lifespan, compared to control animals treated with the ALA solvent alone, with the maximal lifespan observed at the 5 mm dose (Fig. 1B and Table S1, Supporting information). In multiple trials, this concentration consistently produced a ~30% increase in mean lifespan. The effects of ALA on lifespan were observed in both the TJ1060 (*spe‐9; rrf‐3*) strain which is genetically sterile (Fig. 1B), and also in wild‐type N2 worms that were sterilized via the use of fluorodeoxyuridine (FUDR; Fig. 1C).

### ALA treatment activates the nhr‐49/PPARα transcription factor

To identify potential mechanisms by which ALA treatment can increase worm lifespan, we isolated RNA from ten separate pairs of ALA‐treated and control animals and used RNA‐seq to examine changes in gene expression. We identified 64 genes that showed differential expression in the ALA‐treated animals (Appendix S1 and Table S2, Supporting information). Analysis of the upregulated genes using the DAVID program demonstrated the strong enrichment for genes involved in fatty acid metabolism with a 47‐fold enrichment in these genes (false discovery rate 0.002%; Table S3, Supporting information). Within this class, the genes involved in fatty acid degradation showed the greatest enrichment (27‐fold enrichment with a false discovery rate 0.17%; Table S3, Supporting information). Included in the group of fatty acid degradation genes identified were *acs‐2*,* acs‐7*, and *hacd‐1*. Both *acs‐2* and *acs‐7* encode members of the acyl‐CoA synthase family which catalyze the conjugation of fatty acids to coenzyme A, which is an essential first step in the import of fatty acids into the mitochondria and then their conversion into energy via β‐oxidation. The *hacd‐1* gene encodes a member of the 3‐hydroxyacyl‐CoA dehydrogenase family which catalyzes the third reaction in the β‐oxidation process. Additionally, both *acs‐2* and *hacd‐1* have been shown to be regulated by the *nhr‐49*/PPARα nuclear hormone receptor (Van Gilst *et al*., 2005; Ratnappan *et al*., 2014). Furthermore, by manual review of the gene lists, we found that the *C48B4.1/acox‐1.5* and *F59F4.1/acox‐1.6* genes are upregulated in the ALA‐treated worms (Table S2, Supporting information). Both genes encode homologs of the vertebrate ACOX1 peroxisomal acyl‐coenzyme A oxidase enzyme which is similarly upregulated in mice following the consumption of dietary ω‐3 fatty acids and is under the control of the PPARα transcription factor (Ren *et al*., 1997). Together, these findings suggest that a major effect of ALA treatment was the activation of lipid metabolism pathways to perhaps promote the metabolism of ALA via β‐oxidation.

To test the role of β‐oxidation in the lifespan extension produced by ALA treatment, we used *acs‐2‐* and *acs‐7* RNAi‐treated worms for lifespan studies. While we found that *acs‐2* and *acs‐7* RNAi alone had little effect on lifespan following ALA treatment, treatment with a combination of the two RNAi clones did selectively reduce the lifespan of the ALA‐treated worms (Fig. 1E). This experiment may also underestimate the effects of inhibiting *acs‐2* and *acs‐7* as RNAi treatment was only performed during larval development (see Experimental Procedures). Regardless, these findings support a role for an increase in β‐oxidation contributing to the effects of ALA on longevity, but they also could suggest a fair degree of redundancy among the *acs* genes induced by ALA with *acs‐2* and *acs‐7* along with other *acs* genes likely being able to compensate for a lack of specific family members.

We then examined the regulation of *acs‐2* expression via the use of an *acs‐2p::GFP* reporter gene (Burkewitz *et al*., 2015). Treatment of worms carrying the reporter with ALA produced an increase in fluorescence, particularly in the worm intestine (Fig. 1D,F). The increase in GFP fluorescence was produced via the activation of *nhr‐49*, because treatment with *nhr‐49* RNAi blocked the increase produced by ALA treatment (Fig. 1D,F). This activation of *nhr‐49* also played an important role in the increase in lifespan produced by ALA treatment because *nhr‐49* RNAi also greatly reduced the effects of ALA treatment on lifespan (Fig. 1G and Table S1, Supporting information).

### ALA treatment activates the skn‐1/Nrf2 transcription factor

**Figure 2:**
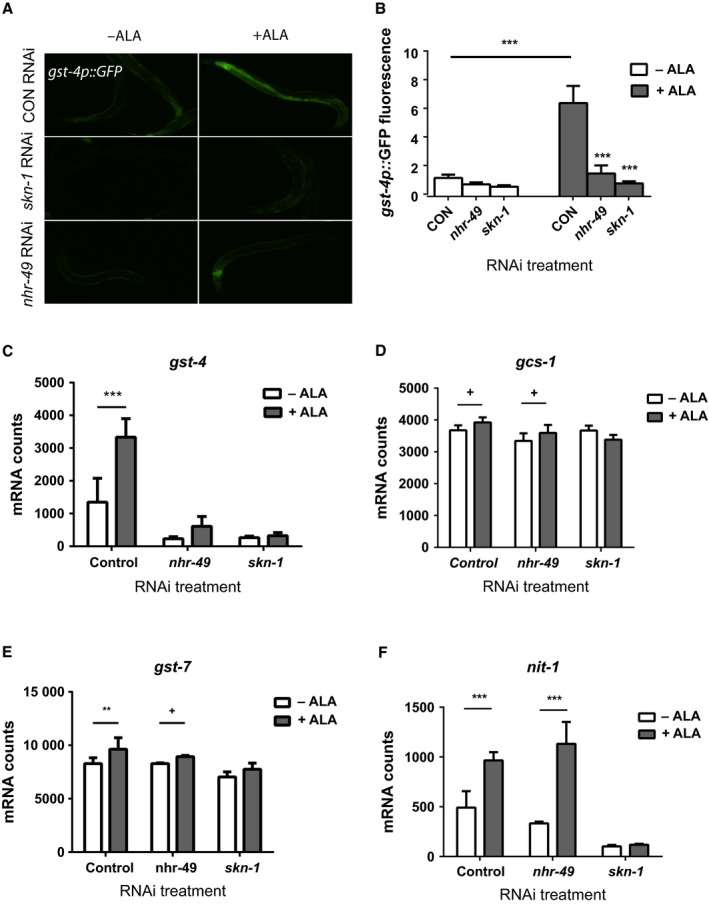
α‐linolenic acid (ALA) treatment activates the *skn‐1*/Nrf2 transcription factor. Treatment of worms carrying a *gst‐4p::GFP* transgene with 5 mm ALA leads to an increase in GFP expression as shown by fluorescence microscopy (A) or quantitation of the GFP fluorescence in the images using the ImageJ program (B). The increased GFP expression produced by ALA treatment requires both *nhr‐49* and *skn‐1* as either RNAi treatment blocks the increase. *N* > 6 for all RNAi and ALA treatment combinations. *** represents *P* < 0.001 for control RNAi –ALA vs. Control RNAi +ALA,* nhr‐49* RNAi +ALA vs. Control RNAi +ALA, and *skn‐1* RNAi +ALA vs. Control RNAi +ALA. The measurement of gene expression via Nanostring also shows an increase in *gst‐4* expression (C) as well as the oxidative stress response genes *gcs‐1* (D), *gst‐7* (E), and *nit‐1* (F). However, only the increased expression of *gst‐4* (C) is inhibited by *nhr‐49* RNAi whereas increases in expression for *gcs‐1* (D), *gst‐7* (E), and *nit‐1* (F) still occur. In contrast, *skn‐1* RNAi treatment blocks the increased expression of all of these genes. *N* = 4 for all Nanostring experiments. *** represents *P* < 0.001, ** represents *P* < 0.01 and + represents 0.05 < *P* < 0.1.

**Figure 3:**
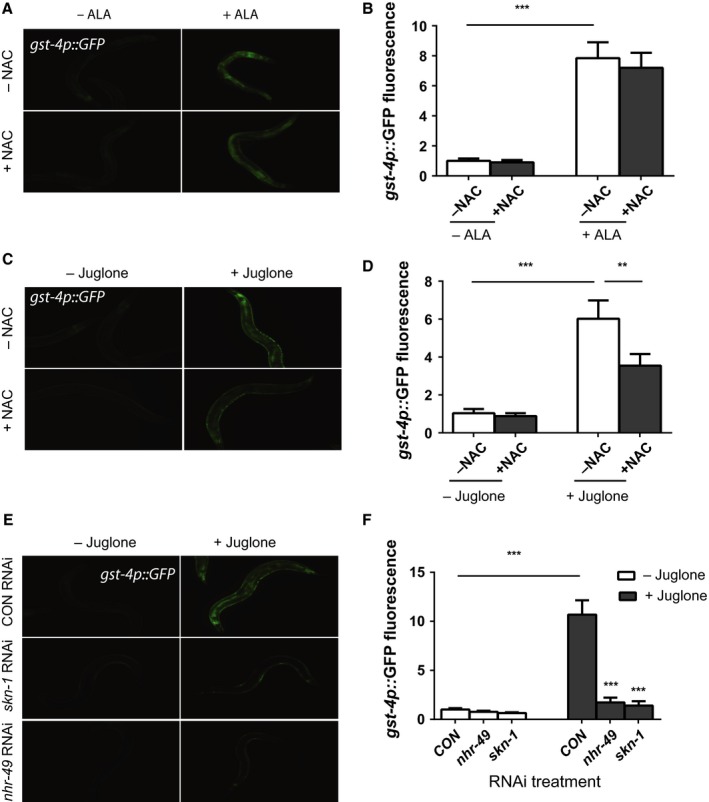
Control of *gst‐4* expression by α‐linolenic acid (ALA) and oxidative stress. ALA treatment activates the expression of the *gst‐4p::GFP* reporter as shown by fluorescence microscopy (A top panels) and quantitation of GFP fluorescence (B). *N* = 6 and *** represents *P* < 0.001 for ‐NAC ‐ ALA vs. −NAC +ALA. However, the effects of ALA on reporter activation do not involve the production of reactive oxygen species (ROS) because the activation is not blocked by pretreatment with N‐acetylcysteine (NAC) (A bottom panels and B). *N* = 6 and *P* = 0.31 ALA treatment +/− NAC. In contrast, NAC treatment is able to attenuate the activation of the *gst‐4p::GFP* reporter produced by oxidative stress produced by juglone treatment as shown by fluorescence microscopy (C) and quantitation of GFP fluorescence (D). *N* = 6 for all treatments. *** represents *P* < 0.0001 by *t*‐test and ** represents *P* = 0.0006 by *t*‐test. (E) After exposure to oxidative stress produced by juglone treatment, both the *skn‐1*/Nrf2 transcription factor and the *nhr‐49*/PPARα gene are required for the activation of the *gst‐4p::GFP* reporter as shown by fluorescence microscopy (E) and quantitation of GFP fluorescence (F). *N* = 6 and *** represents *P* < 0.001 for Control RNAi ‐ juglone vs. Control RNAi +juglone, Control RNAi + juglone vs. *nhr‐49* RNAi +juglone and Control RNAi + juglone vs. s*kn‐1* RNAi +juglone.

Recent work has demonstrated an important role for the *skn‐1* transcription factor in the increased lifespan of the *glp‐1* mutant animals (Steinbaugh *et al*., 2015). To examine whether *skn‐1* could also be activated by ALA treatment, we treated worms carrying a *gst‐4p::GFP* transgene with 5 mm ALA and observed increases in fluorescence beginning within hours of treatment (Fig. 2A,B). The activation of *gst‐4* expression was mediated by *skn‐1* because we found that *skn‐1* RNAi blocked the increase in fluorescence following ALA treatment (Fig. 2A,B). We also observed the activation of other oxidative stress response genes including *gcs‐1*,* gst‐7*, and *nit‐1* along with *gst‐4* via the use of gene expression measurement by Nanostring, although the magnitude of the effect varied between the genes with *gst‐4* and *nit‐1* responding, for unclear reasons, more robustly than *gcs‐1* and *gst‐7* (Fig. 2C–F). This could suggest that only a subset of oxidative stress response genes participate in the response to ALA treatment. Surprisingly, the full increase in *gst‐4* expression also required the *nhr‐49* transcription factor because *nhr‐49* RNAi reduced both the basal expression of *gst‐4* and attenuated the increase in expression after ALA treatment (Fig. 2C, where *P* = 0.19 for ALA treated vs. control) as well as the increase in *gst‐4p::GFP* fluorescence (Fig. 2A,B). We hypothesized that the activation of *skn‐1* could be mediated by mitohormesis perhaps due to an increase in β‐oxidation and mitochondrial activity produced by *nhr‐49*. However, treatment of worms with the antioxidant N‐acetylcysteine was unable to block the increase in GFP fluorescence (Fig. 3A,B), while this treatment did attenuate increases due to treatment with the reactive oxygen species (ROS) generator juglone (Fig. 3C,D). This suggested that a generalized increase in ROS production was unlikely to account for the activation of *skn‐1*.

We then asked whether the activation of other oxidative stress response genes depended on the *nhr‐49* transcription factor. We found that *nhr‐49* RNAi selectively reduced the expression of *gst‐4* compared to *gcs‐1*,* gst‐7*, and *nit‐1* which showed little change after *nhr‐49* RNAi treatment (Fig. 2C–F). Based on this finding, we hypothesized that *gst‐4* could be regulated by *nhr‐49* following exposure to other forms of oxidative stress. To test this possibility, we used juglone to activate the *gst‐4::GFP* transgene, and as expected, we found that *skn‐1* RNAi blocked the increase in GFP expression (Fig. 3E,F), but we also found that *nhr‐49* RNAi potently blocked these increases (Fig. 3E,F). Hence, the expression of *gst‐4* expression following either ALA treatment or direct exposure to oxidative stress depends on both *skn‐1* and *nhr‐49*, and *gst‐4* may represent a novel target gene for *nhr‐49*. If so, this could suggest that there is an intimate linkage of metabolism and the oxidative stress response.

**Figure 4:**
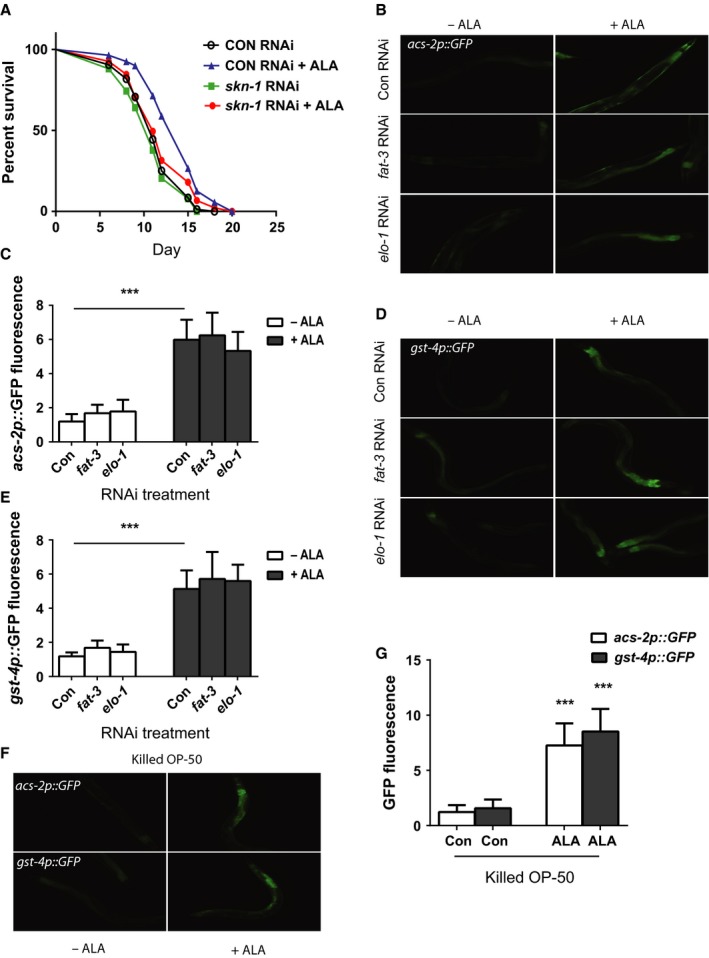
α‐linolenic acid (ALA) treatment increases worm lifespan via *skn‐1*/Nrf2. (A) The increase in worm lifespan produced by treatment with 5 mm ALA requires *skn‐1* because animals treated with *skn‐1* RNAi show no increase in lifespan following ALA treatment. *N* = 83 for control RNAi ‐ALA, 95 for control RNAi +ALA, 75 for *skn‐1* RNAi ‐ALA, 56 for *skn‐1* RNAi +ALA. *P* < 0.0001 for control RNAi ‐ALA vs. +ALA by log‐rank test, and *P* = NS for *skn‐1* RNAi ‐ALA vs. +ALA by log‐rank test. (B) The activation of the *acs‐2p::GFP* reporter after ALA treatment is not reduced by blocking the synthesis of longer‐chain ω‐3 fatty acids with *fat‐3* or *elo‐1* RNAi as shown by digital imaging or the quantification of GFP fluorescence (C). *N* = 6 for all treatments. *** represents *P* < 0.0001 by *t‐*test. (D) The activation of the *gst‐4p::GFP* reporter after ALA treatment is also not blocked by *fat‐3* or *elo‐1* RNAi treatment as shown by digital imaging or quantification of GFP fluorescence (E). *N* = 6 for all treatments. *** represents *P* < 0.0001 by *t‐*test. (F) The activation of the *acs‐2p::GFP* and *gst‐4p::GFP* reporters still occurs when worms are fed ALA with heat‐killed bacteria as shown by digital imaging or the quantification of GFP fluorescence (G). *N* = 6 for all treatments. *** represents *P* < 0.0001 by *t*‐test.

To test whether the increased lifespan of the ALA‐treated animals requires the activity of *skn‐1*, we treated worms with *skn‐1* RNAi. The RNAi treatment largely blocked the effects of ALA on lifespan which demonstrates important roles for both *skn‐1* and *nhr‐49* in promoting longevity in response to ALA treatment (Fig. 4A and Table S1, Supporting information).

### ALA does not require conversion to longer‐chain fatty acids or bacterial metabolism

*Caenorhabditis elegans* has the ability to synthesize a number of ω‐3 fatty acids that are essential in mammals including eicosapentaenoic acid (EPA) and docosahexaenoic acid (DHA) acid (Watts & Browse, 2002). Alternatively, the bacteria used as a food source for the worms are also metabolically active and could promote the conversion of ALA into other lipids or a metabolic intermediate that are biologically active. As a result, we tested whether the elongation of ALA into other fatty acids in *C. elegans* or the metabolism of ALA by bacteria altered the effects of ALA on the expression of *acs‐2p::GFP* or *gst‐4p::GFP*. We blocked the elongation of ALA in worms via the use of either *fat‐3* or *elo‐1* RNAi. Both *fat‐3*, which encodes a Δ6 desaturase, and *elo‐1*, which encodes a fatty acid elongase, are essential for the formation of polyunsaturated fatty acids that are twenty or more carbons long (Watts & Browse, 2002). We found that neither treatment with *fat‐3* nor *elo‐1* RNAi reduced the activation of either reporter (Fig. 4B–E) which suggests that ALA instead of a longer‐chain ω‐3 fatty acid is responsible for the activation of these reporters. We also spotted ALA‐containing plates with heat‐killed OP50‐1 bacteria to test the role of bacterial metabolism in the effects of ALA. We found that both reporters can still be activated in worms fed killed bacteria (Fig. 4F,G). Together, these data suggest that ALA alone, and not a longer‐chain ω‐3 fatty acid, contributes to the activation of *nhr‐49*, and also that the activation of *nhr‐49* and *skn‐1* does not require the conversion of ALA into other fatty acids or metabolites via bacterial metabolism.

### ALA is oxidized to oxylipins

**Figure 5:**
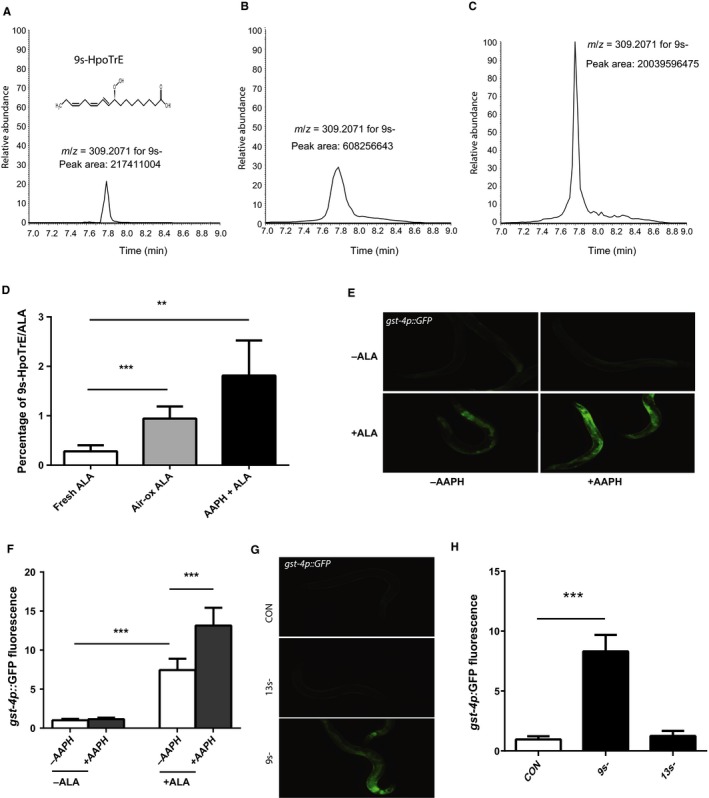
Oxidation of α‐linolenic acid (ALA) activates *skn‐1* via the production of oxylipins. The exposure of ALA to either oxygen in room air (B) or to the oxidizer 2,2′‐Azobis(2‐methylpropionamidine) dihydrochloride (AAPH) (C) leads to the accumulation of the oxylipin 9S‐hydroperoxy‐10E,12Z,15Z‐octadecatrienoic acid (9(S)‐HpOTrE) as shown via the use of high‐performance liquid chromatography followed by mass spectrometry compared to a freshly opened aliquot of ALA (A). Shown in (A) is the structure of 9(S)‐HpOTrE as an inset which shows the shift in desaturations and presence of a peroxide group compared to ALA. As a result of the oxidation of the ALA to 9(S)‐HpOTrE, the percentage of each sample that is 9(S)‐HpOTrE compared to ALA also increases from 0.3% to almost 1.8% (D). *N* = 5 for all samples. ***P < 0.0006 for fresh ALA vs. air‐oxidized ALA. *P* = 0.0078 for fresh ALA vs. AAPH‐oxidized ALA by *t*‐test. (E) The treatment of worms with the AAPH‐oxidized ALA leads to a greater increase in the expression of the *gst‐4p::GFP* reporter (E bottom right) compared to the treatment of worms with either the unoxidized ALA (E bottom left) or the vehicle solution treated with AAPH and then neutralized in a similar fashion (E top right). (F) These effects are also seen when the fluorescence in additional images are measured by the ImageJ program. *N* = 6 and *** represents *P* < 0.001 for −AAPH −ALA vs. −AAPH +ALA and −AAPH +ALA vs. +AAPH +ALA. (G) The treatment of worms carrying the *gst‐4p::GFP* reporter with 9(S)‐HpOTrE but not the isomer 13(S)‐HpOTrE at a concentration of 0.2 mm leads to an increase in GFP fluorescence. (H) Quantification of GFP fluorescence from animals treated with the specified oxylipin and then imaged as in (G). *N* = 6 and *** represents *P* < 0.001 for control vs. 9(S)‐HpOTrE treatment.

The ω‐3 fatty acids are subject to chemical and enzymatic modifications involving reactions such as hydroxylation and peroxidation to produce a class of molecules termed oxylipins (Mosblech *et al*., 2009; Gabbs *et al*., 2015). Often, these reactions give rise to molecules, such as prostaglandins, that are biologically active and have specific effects that are distinct from the parent lipid. We noted that the effects of ALA seemed to differ based on the age of the stock and age of the ALA‐containing NGA plates used for study. As a result, we hypothesized that ALA may undergo oxidation and that one or more oxylipins could contribute to the effects of ALA that we observed. Prior work suggested that ALA undergoes rapid oxidation after contact with air, and the predominant oxylipin formed by this reaction is 9S‐hydroperoxy‐10E,12Z,15Z‐octadecatrienoic acid (9(S)‐HpOTrE; Yan *et al*., 2015). As a result, we tested whether the exposure of fresh ALA to air leads to the production of 9(S)‐HpOTrE via the use of liquid chromatography followed by mass spectrometry (LC‐MS). We found that this oxylipin formed fairly rapidly and accumulated in the air‐exposed ALA (Fig. 5A,B,D). We then treated ALA with the chemical oxidizer 2,2′‐Azobis(2‐methylpropionamidine) dihydrochloride (AAPH), because prior work has shown this compound to effectively convert ω‐3 fatty acids into oxylipins (Gao *et al*., 2006). This resulted in an even greater accumulation of 9(S)‐HpOTrE compared to air exposure (Fig. 5C,D).

To determine whether oxylipins could contribute to the biologic effects of ALA, we treated worms carrying the *gst‐4p::GFP* reporter with ALA that had been oxidized with AAPH and then neutralized with ascorbic acid. We found that exposure to the neutralized AAPH had no effect on GFP expression (Fig. 5E,F) whereas the treatment of ALA with AAPH further enhanced the activation of the reporter transgene over the level produced by the untreated ALA (Fig. 5E,F). We then purchased the purified oxylipins 9(S)‐HpOTrE and the closely related isomer 13(S)‐HpOTrE to directly test whether these oxylipins lead to the activation of oxidative stress response genes. Treatment of worms with 9(S)‐HpOTrE, but not 13(S)‐HpOTrE, at a concentration of 0.2 mm leads to increased expression of the *gst‐4p::GFP* reporter (Fig. 5G,H). The 0.2 mm concentration was selected to recreate the concentration of 9(S)‐HpOTrE found in the chemically oxidized ALA samples as measured by mass spectrometry. While the mechanisms accounting for the specificity of 9(S)‐HpOTrE compared to 13(S)‐HpOTrE are unclear, this finding suggests that individual oxylipins could be formed by the oxidation of ALA and contribute to specific aspects of the effects of ALA on the treated worms.

### 9(S)‐HpOTrE activates skn‐1

**Figure 6:**
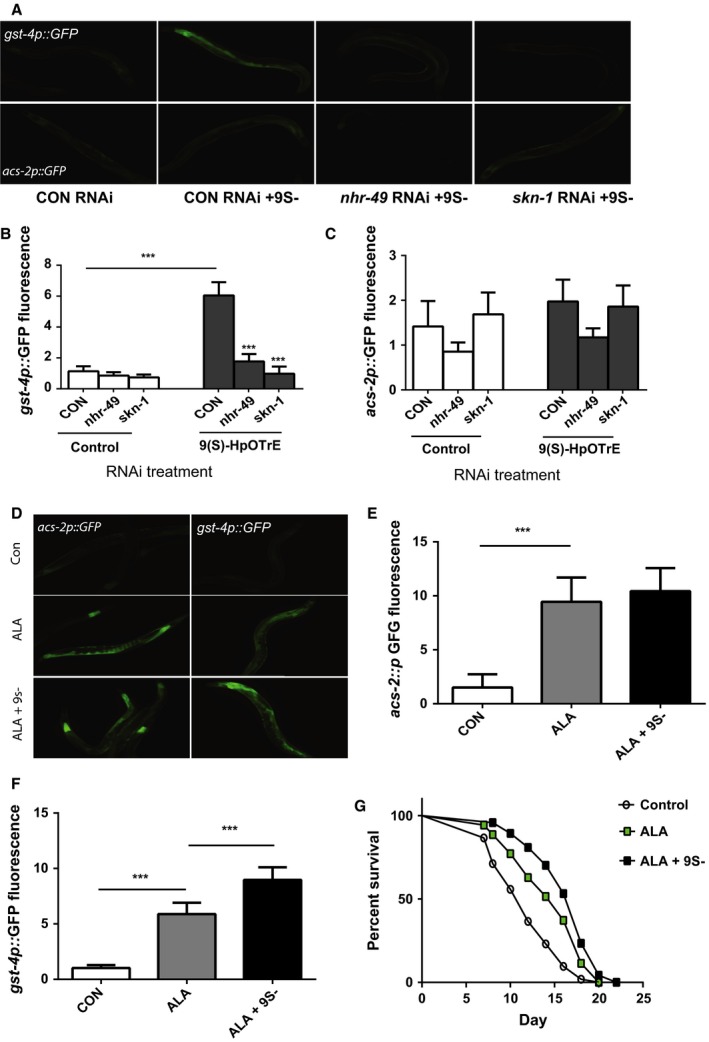
The oxylipin 9S‐hydroperoxy‐10E,12Z,15Z‐octadecatrienoic acid (9(S)‐HpOTrE) acts via the *skn‐1* transcription factor and enhances the effects of α‐linolenic acid (ALA) on worm lifespan. (A) The *gst‐4p::GFP* reporter is activated by 9(S)‐HpOTrE and requires the *nhr‐49* and *skn‐1* transcription factors (top panels) whereas 9(S)‐HpOTrE has little effect on the expression of the *acs‐2p::GFP* reporter (bottom panels). These effects are also seen in larger groups of worms that are similarly treated and imaged with the images being used for quantification of change in GFP fluorescence for the *gst‐4p::GFP* (B) and *acs‐2p::GFP* (C) reporters. For (B), *N* = 6 for all treatments. *** represents *P* < 0.0001 by *t*‐test. For (C), *N* = 6 for all treatments. *P* = NS for control vs. 9(S)‐HpOTrE treatment of the *acs‐2p::GFP* reporter. (D) The supplementation of a freshly opened ALA stock does not affect the expression of the lipid metabolism gene *acs‐2* (A left panels) but produces a further increase in the expression of *gst‐4* (right panels). These effects are also seen in larger groups of worms that are similarly treated and imaged with the images being used for quantitation of changes in GFP fluorescence for the *acs‐2p::GFP* (E) and *gst‐4p::GFP* reporters (F). For (E), *N* = 6 for each treatment and *** represents *P* < 0.001 for control vs. ALA. *P* = 0.45 for ALA vs. ALA + 9(S)‐HpOTrE. For (F), *N* = 6 for each treatment and *** represents *P* < 0.001 for control vs. ALA and ALA vs. ALA + 9(S)‐HpOTrE. (G) Treatment of worms with ALA‐supplemented 9(S)‐HpOTrE leads to a greater increase in worm lifespan compared to the treatment of worms with freshly prepared ALA alone *N* = 58 for control treatment, 39 for ALA alone, and 50 for ALA + 9(S)‐HpOTrE. *P* = 0.0013 for control vs. ALA alone by log‐rank test, and *P* = 0.04 for ALA vs. ALA + 9(S)‐HpOTrE by log‐rank test.

Our data showed that 9(S)‐HpOTrE can activate the *gst‐4p::GFP* reporter, but we asked whether 9(S)‐HpOTrE acted via the *skn‐1* transcription factor to activate *gst‐4* expression, and whether due to its structural similarity to fatty acids (Fig. 5A inset), 9(S)‐HpOTrE could activate the expression of *nhr‐49* target genes such as *acs‐2*. We treated worms carrying the *gst‐4p::GFP* reporter with control, *skn‐1*, and *nhr‐49* RNAi, and we found that the increase in GFP expression could be strongly reduced with either *skn‐1* and *nhr‐49* RNAi (Fig. 6A,B). Despite the involvement of *nhr‐49* in the control of *gst‐4* expression, 9(S)‐HpOTrE treatment had little effect on the expression of the *acs‐2p::GFP* reporter (Fig. 6A,C). Also, the treatment of worms carrying the *acs‐2p::GFP* reporter with *nhr‐49* or *skn‐1* RNAi had little effect on GFP expression, except that *nhr‐49* RNAi reduced the basal expression of the reporter. Hence, while *nhr‐49* is involved either directly or indirectly in the control of *gst‐4* expression, our results suggests that 9(S)‐HpOTrE primarily acts via the activation of *skn‐1*.

### 9(S)‐HpOTrE enhances the effects of ALA on lifespan

The dual role for *nhr‐49* and *skn‐1* for the enhanced lifespan produced by ALA treatment could suggest that both the ALA parent lipid and the oxidation to oxylipins like 9(S)‐HpOTrE have synergistic effects on gene expression and lifespan. This would also be consistent with our experiments suggesting that 9(S)‐HpOTrE has little effect alone on the lifespan of treated worms (WQ and ALF unpublished data). To test the possibility of a synergistic effect, we treated worms with either a freshly prepared stock of ALA or the same ALA stock that had been supplemented with 0.2 mm 9(S)‐HpOTrE. Both ALA and the mixture of ALA and 9(S)‐HpOTrE produced increases in the expression of the *acs‐2p::GFP* reporter and the *gst‐4p::GFP* reporter (Fig. 6D). However, the effects of each treatment on the *acs‐2p::GFP* reporter differed little (Fig. 6D,E), whereas treatment with ALA and 9(S)‐HpOTrE produced a much greater increase in the expression of the *gst‐4::GFP* reporter compared to ALA treatment alone (Fig. 6D,F). Similarly, the addition of 9(S)‐HpOTrE to the fresh ALA produced a greater effect on lifespan than did the ALA treatment alone (Fig. 6G). These findings suggest that the synergy between ALA and oxylipins, such as 9(S)‐HpOTrE, plays a key role in the effects of this ω‐3 fatty acid on lifespan.

## Discussion

### ALA increases *C. elegans* lifespan

Our data demonstrate that the ω‐3 fatty acid α‐linolenic acid (ALA) is able to increase the lifespan of *C. elegans* via the activation of the NHR‐49/PPARα and SKN‐1/Nrf2 transcription factors. At least part of the role for NHR‐49 involves the activation of genes involved in the metabolism of fatty acids, and in the particular, an increase in the expression of genes involved in the β‐oxidation of free fatty acids. This process promotes the generation of energy from the fatty acids via the use of the tricarboxylic acid (TCA) cycle and mitochondrial respiration. Increases in mitochondrial respiration have been previously linked to lifespan increases through a process termed mitohormesis, which involves benefits directly produced by the low levels of oxidative stress generated by enhanced mitochondrial activity (Schulz *et al*., 2007). Consistent with a possible role for mitohormesis in the ALA‐treated animals, we do see the activation of oxidative stress responses and a critical role for the SKN‐1 transcription factor in the lifespan benefits of ALA treatment (Figs 2 and 4). However, the activation of SKN‐1 activity is largely produced by ALA‐derived oxylipins as opposed to an increase in reactive oxygen species (Figs 3 and 5). Consistently, the oxidation of EPA and DHA also leads to the production of oxylipins that react with thiols in the Keap1 protein that normally inhibits the SKN‐1 ortholog Nrf2 by retaining the protein in the cytoplasm (Gao *et al*., 2007). This leads to the activation of Nrf2 both in culture cells and *in vivo*, and contributes to the benefits of ω‐3 fatty acid supplementation seen in experimental stroke models (Gao *et al*., 2007; Zhang *et al*., 2014). While this does not exclude a beneficial role for mitohormesis in the effects of ALA, it does suggest that the oxylipin‐mediated activation of oxidative stress responses acts to enhance the positive effects of enhanced lipid metabolism.

ALA also likely plays a physiologic role in the effects of germ line signaling on aging in worms because the loss of germ line stem cells results in an increase in fat synthesis with ALA being one of the lipids showing enhanced production (Amrit *et al*., 2016). Coupled with the increase in lipid synthesis is also an increase in fat metabolism which suggests that a metabolic shift toward the use of lipids as an energy source occurs in these animals (Ratnappan *et al*., 2014). Consistent with a possible role for ALA in the response to changes in germ line signaling, the enhanced lifespan of the germ line‐less animals also depends upon both NHR‐49/PPARα and SKN‐1/Nrf2 (Ratnappan *et al*., 2014; Steinbaugh *et al*., 2015). However, the response to changes in germ line signaling is more complex than solely being a response to changes in ALA levels as other transcription factors, like the DAF‐16/FOXO, are also essential for the enhanced longevity of these animals (reviewed in Ghazi, 2013). Additionally, the production and/or release of other lipids in the germ line‐less animals, such as oleic acid and ω‐6 fatty acids, also act to retard aging (Goudeau *et al*., 2011; O'Rourke *et al*., 2013).

### The biologic effects of ω‐3 fatty acids involve oxylipins

Many of the biologically relevant ω‐3 fatty acids are chemically reactive and can undergo both spontaneous and enzymatically mediated oxidation reactions (Mosblech *et al*., 2009; Gabbs *et al*., 2015). These chemical reactions lead to the production of one to a potentially large number of distinct oxylipin molecules. Importantly, these oxylipins often do not simply represent subtly altered versions of the parent lipid which might have changes in water solubility or other chemical features, but instead, the oxylipins represent distinct and often biologically active molecules that can be recognized by unique receptors or promote biologic effects that are quite different than the actions of the parent compound (Mosblech *et al*., 2009; Gabbs *et al*., 2015). For example, the beneficial effects of fish consumption on cardiovascular outcomes have been hypothesized to involve the production of beneficial eicosanoids via the enzyme‐mediated oxidation of the ω‐3 fatty acids EPA and DHA (von Schacky, 1987). Our data demonstrate a novel role for cooperative interactions between both the parent ω‐3 fatty acid and an oxylipin, 9(S)‐HpOTrE, produced via the nonenzymatic oxidation of the lipid. This oxylipin can enhance the effects of ALA through the activation of complementary biologic mechanisms.

The striking differences between dietary and supplement‐derived ω‐3 fatty acids on health outcomes, with dietary sources showing benefits and supplements being largely ineffective, have prompted a search for underlying reasons. Among the factors that have been identified are critical differences in the bioavailability and lipid structures between ω‐3 fatty acid rich foods and supplements consisting of purified oils that contain high levels of ω‐3 fatty acids (Schuchardt & Hahn, 2013). An additional possibility is that the levels and diversity of oxylipins may differ between foods and supplements, and the lack of oxylipins could contribute to the reduced benefits produced by the supplements.

## Experimental procedures

### Strains

The *C*. *elegans* strains TJ1060 (*spe‐9(hc88); fer‐15(b26*); Fabian & Johnson, 1995), CL2166 (*dvIs19[pAF15(gst‐4::GFP::NLS)]*; Link & Johnson, 2002), and WBM170 (*wbmEx57 [acs‐2p::GFP + rol‐6(su1006)]*; Burkewitz *et al*., 2015) were provided by the Caenorhabditis Genetics Center (Minneapolis, MN, USA) which is supported by NIH funding. All strains were propagated on standard nematode growth agar (NGA) plates containing streptomycin (200 μg/mL) and spotted with OP50‐1, as previously described (Sulston & Horvitz, 1988).

### Chemical treatments

To treat worms with ALA, aliquots from a 660 mm stock of α‐Linolenic Acid (Cayman Chemical, Ann Arbor, Michigan), which was dissolved in a surfactant and stabilization solution containing vitamin E, Tween 80, and ethanol, were added to molten NGA to cast the ALA in to the prepared plates at the desired final concentration. As a control, equal volumes of the same solution lacking ALA were added to a separate batch of molten NGA plates poured at the same time. Plates were then dried at room temperature and spotted with OP50‐1. Due to observed development defects produced by ALA, animals were grown to day 1 of adulthood on standard media and then transferred to plates containing ALA or the vehicle‐only control. ALA or control treatment continued for the remainder of the imaging or lifespan study. Due to the potential for ALA to become oxidized during storage, all plates were used with 6 weeks. For some experiments, ‘fresh’ ALA plates were those prepared and used within 1 week, while ‘oxidized’ ALA plates were allowed to oxidize at 4 °C for 1 month prior to use. To produce chemically oxidized ALA, an aliquot of the ALA mixture was incubated with an equal volume of 20 mm 2,2′‐Azobis(2‐methylpropionamidine) dihydrochloride (AAPH; Sigma‐Aldrich, St. Louis, MO, USA) dissolved in PBS solution at 37 °C overnight (Gao *et al*., 2006). The AAPH was then neutralized by adding 10 mm Vitamin C (Lotito & Fraga, 2000). Aliquots of the oxidized ALA, or the AAPH‐oxidized surfactant mixture as a control, were then added to molten NGA media.

For oxylipin treatments, 9S‐hydroperoxy‐10E,12Z,15Z‐octadecatrienoic acid (9(S)‐HpOTrE) and 13S‐hydroperoxy‐9Z,11E,15Z‐octadecatrienoic acid (13(S)‐HpOTrE) were purchased from Cayman Chemical (Ann Arbor, Michigan) and dissolved in 100% ethanol at 2 mm concentration. The chemicals, or an equal volume of 100% ethanol as a control, were then added to molten NGA media to produce plates with a final compound concentration of 0.2 mm. For combined ALA and 9(S)‐HpOTrE treatments, 9(S)‐HpOTrE, or an equal volume of 100% ethanol, was added along with ALA to molten NGA to give a final concentration of 0.2 mm 9(S)‐HpOTrE and 5 mm ALA.

Treatment of worms with 38 μm juglone was performed in M9 media as previously described (Ferguson *et al*., 2010). The worms were treated for 1 h before being rinsed and returned to NGA plates for 8 h prior to imaging.

### Fluorescence imaging

Effects of RNAi and compound treatments on GFP reporters were assessed by fluorescence microscopy using a Nikon Eclipse Ti inverted microscope and CoolSNAP ES2 digital camera as previously described (Keith *et al*., 2016). GFP fluorescence intensity was quantified using ImageJ (Abramoff *et al*., 2004). Statistical testing was performed using unpaired *t*‐tests with the Prism6 software (Graphpad Software, La Jolla, CA, USA). A minimum of two trials with comparable results were performed for all imaging experiments.

### Lifespan studies

Lifespan assays were conducted at 25 °C using the TJ1060 (*spe‐9(hc88); fer‐15(b26*)) strain, except for Fig. 1C that used wild‐type N2 animals sterilized via treatment with 50 μm fluordeoxyuridine, which is sterile at this temperature (Fabian & Johnson, 1995). This strain was propagated at 16 °C before being synchronized by hypochlorite treatment. The eggs were then allowed to grow to adulthood at 25 °C on NGA plates. On day 1 of adulthood, 40–50 worms were transferred to two or three NGA plates containing either ALA or the appropriate control and spotted with OP50‐1. Animals were scored every 1–2 day for death or phenotypes, such as bagging, that result in censoring. Prism6 (Graphpad Software, La Jolla, CA, USA) was used to generate graphs and perform log‐rank testing for curve comparisons, Stata 14 (Stata Corp, College Station, TX, USA) was used to create life tables and calculate mean survival. A minimum of two trials with comparable results were performed for all lifespan studies. Data from each lifespan are included in Table S1 (Supporting information).

## Funding

This work was supported by funds from the South Texas VA Healthcare System, National Institute of Aging grants AG013319 and AG044768, and National Institute of Environmental Health Sciences grant ES017761 to ALF.

## Author contributions

WQ, GEG, HD, JAM, AMM, and ALF conceived and designed the experiments. WQ, XG, and ALF performed the experiments. GEG, HD, JAM contributed reagents/materials/analysis tools. WQ, XG, JAM, AMM, and ALF analyzed the data. WQ and ALF wrote the paper.

## Conflict of interest

None declared.

## Supporting information


**Table S1** Mean survival and lifetable data for lifespan experiments.

**Table S2** Genes identified as being regulated by α‐linolenic acid (ALA) treatment via the use of RNA‐seq.

**Table S3** Analysis of differentially expressed genes via the use of the DAVID program.

**Appendix S1** Additional experimental procedures.

## References

[acel12651-bib-0001] Abramoff MD , Magalhaes PJ , Ram SJ (2004) Image Processing with ImageJ, Biophotonics International 11, 36–42.

[acel12651-bib-0002] Amrit FR , Steenkiste EM , Ratnappan R , Chen SW , McClendon TB , Kostka D , Yanowitz J , Olsen CP , Ghazi A (2016) DAF‐16 and TCER‐1 facilitate adaptation to germline loss by restoring lipid homeostasis and repressing reproductive physiology in *C. elegans* . PLoS Genet. 12, e1005788. 26862916 10.1371/journal.pgen.1005788 PMC4749232

[acel12651-bib-0003] Burkewitz K , Morantte I , Weir HJ , Yeo R , Zhang Y , Huynh FK , Ilkayeva OR , Hirschey MD , Grant AR , Mair WB (2015) Neuronal CRTC‐1 governs systemic mitochondrial metabolism and lifespan via a catecholamine signal. Cell 160, 842–855. 25723162 10.1016/j.cell.2015.02.004 PMC4392909

[acel12651-bib-0004] Chowdhury R , Warnakula S , Kunutsor S , Crowe F , Ward HA , Johnson L , Franco OH , Butterworth AS , Forouhi NG , Thompson SG , Khaw KT , Mozaffarian D , Danesh J , Di Angelantonio E (2014) Association of dietary, circulating, and supplement fatty acids with coronary risk: a systematic review and meta‐analysis. Ann. Intern. Med. 160, 398–406. 24723079 10.7326/M13-1788

[acel12651-bib-0005] Fabian TJ , Johnson TE (1995) Identification genes that are differentially expressed during aging in *Caenorhabditis elegans* . J. Gerontol. A Biol. Sci. Med. Sci. 50, B245–B253. 7671015 10.1093/gerona/50a.5.b245

[acel12651-bib-0006] Ferguson AA , Springer MG , Fisher AL (2010) skn‐1‐Dependent and ‐independent regulation of aip‐1 expression following metabolic stress in *Caenorhabditis elegans* . Mol. Cell. Biol. 30, 2651–2667. 20351174 10.1128/MCB.01340-09 PMC2876512

[acel12651-bib-0007] Folick A , Oakley HD , Yu Y , Armstrong EH , Kumari M , Sanor L , Moore DD , Ortlund EA , Zechner R , Wang MC (2015) Aging. Lysosomal signaling molecules regulate longevity in *Caenorhabditis elegans* . Science 347, 83–86. 25554789 10.1126/science.1258857 PMC4425353

[acel12651-bib-0008] Gabbs M , Leng S , Devassy JG , Monirujjaman M , Aukema HM (2015) Advances in our understanding of oxylipins derived from dietary PUFAs. Adv. Nutr. 6, 513–540. 26374175 10.3945/an.114.007732 PMC4561827

[acel12651-bib-0009] Gao L , Yin H , Milne GL , Porter NA , Morrow JD (2006) Formation of F‐ring isoprostane‐like compounds (F3‐isoprostanes) *in vivo* from eicosapentaenoic acid. J. Biol. Chem. 281, 14092–14099. 16569632 10.1074/jbc.M601035200

[acel12651-bib-0010] Gao L , Wang J , Sekhar KR , Yin H , Yared NF , Schneider SN , Sasi S , Dalton TP , Anderson ME , Chan JY , Morrow JD , Freeman ML (2007) Novel n‐3 fatty acid oxidation products activate Nrf2 by destabilizing the association between Keap1 and Cullin3. J. Biol. Chem. 282, 2529–2537. 17127771 10.1074/jbc.M607622200

[acel12651-bib-0011] Ghazi A (2013) Transcriptional networks that mediate signals from reproductive tissues to influence lifespan. Genesis 51, 1–15. 22945891 10.1002/dvg.22345

[acel12651-bib-0012] Goudeau J , Bellemin S , Toselli‐Mollereau E , Shamalnasab M , Chen Y , Aguilaniu H (2011) Fatty acid desaturation links germ cell loss to longevity through NHR‐80/HNF4 in *C. elegans* . PLoS Biol. 9, e1000599. 21423649 10.1371/journal.pbio.1000599 PMC3057950

[acel12651-bib-0013] Hsin H , Kenyon C (1999) Signals from the reproductive system regulate the lifespan of *C. elegans* . Nature 399, 362–366. 10360574 10.1038/20694

[acel12651-bib-0014] Keith SA , Maddux SK , Zhong Y , Chinchankar MN , Ferguson AA , Ghazi A , Fisher AL (2016) Graded proteasome dysfunction in *Caenorhabditis elegans* activates an adaptive response involving the conserved SKN‐1 and ELT‐2 transcription factors and the autophagy‐lysosome pathway. PLoS Genet. 12, e1005823. 26828939 10.1371/journal.pgen.1005823 PMC4734690

[acel12651-bib-0015] Lapierre LR , Gelino S , Melendez A , Hansen M (2011) Autophagy and lipid metabolism coordinately modulate life span in germline‐less *C. elegans* . Curr. Biol. 21, 1507–1514. 21906946 10.1016/j.cub.2011.07.042 PMC3191188

[acel12651-bib-0016] Link CD , Johnson CJ (2002) Reporter transgenes for study of oxidant stress in *Caenorhabditis elegans* . Methods Enzymol. 353, 497–505. 12078522 10.1016/s0076-6879(02)53072-x

[acel12651-bib-0017] Lotito SB , Fraga CG (2000) Ascorbate protects (+)‐catechin from oxidation both in a pure chemical system and human plasma. Biol. Res. 33, 151–157. 15693282 10.4067/s0716-97602000000200015

[acel12651-bib-0018] Mosblech A , Feussner I , Heilmann I (2009) Oxylipins: structurally diverse metabolites from fatty acid oxidation. Plant Physiol. Biochem. 47, 511–517. 19167233 10.1016/j.plaphy.2008.12.011

[acel12651-bib-0019] O'Rourke EJ , Soukas AA , Carr CE , Ruvkun G (2009) *Caenorhabditis elegans* major fats are stored in vesicles distinct from lysosome‐related organelles. Cell Metab. 10, 430–435. 19883620 10.1016/j.cmet.2009.10.002 PMC2921818

[acel12651-bib-0020] O'Rourke EJ , Kuballa P , Xavier R , Ruvkun G (2013) omega‐6 Polyunsaturated fatty acids extend life span through the activation of autophagy. Genes Dev. 27, 429–440. 23392608 10.1101/gad.205294.112 PMC3589559

[acel12651-bib-0021] Ratnappan R , Amrit FR , Chen SW , Gill H , Holden K , Ward J , Yamamoto KR , Olsen CP , Ghazi A (2014) Germline signals deploy NHR‐49 to modulate fatty‐acid beta‐oxidation and desaturation in somatic tissues of *C. elegans* . PLoS Genet. 10, e1004829. 25474470 10.1371/journal.pgen.1004829 PMC4256272

[acel12651-bib-0022] Ren B , Thelen AP , Peters JM , Gonzalez FJ , Jump DB (1997) Polyunsaturated fatty acid suppression of hepatic fatty acid synthase and S14 gene expression does not require peroxisome proliferator‐activated receptor α. J. Biol. Chem. 272, 26827–26832. 9341113 10.1074/jbc.272.43.26827

[acel12651-bib-0023] von Schacky C (1987) Prophylaxis of atherosclerosis with marine omega‐3 fatty acids. A comprehensive strategy. Ann. Intern. Med. 107, 890–899. 2825573 10.7326/0003-4819-107-6-890

[acel12651-bib-0024] Schuchardt JP , Hahn A (2013) Bioavailability of long‐chain omega‐3 fatty acids. Prostaglandins Leukot. Essent. Fatty Acids 89, 1–8. 23676322 10.1016/j.plefa.2013.03.010

[acel12651-bib-0025] Schulz TJ , Zarse K , Voigt A , Urban N , Birringer M , Ristow M (2007) Glucose restriction extends *Caenorhabditis elegans* life span by inducing mitochondrial respiration and increasing oxidative stress. Cell Metab. 6, 280–293. 17908557 10.1016/j.cmet.2007.08.011

[acel12651-bib-0026] Steinbaugh MJ , Narasimhan SD , Robida‐Stubbs S , Moronetti Mazzeo LE , Dreyfuss JM , Hourihan JM , Raghavan P , Operana TN , Esmaillie R , Blackwell TK (2015) Lipid‐mediated regulation of SKN‐1/Nrf in response to germ cell absence. eLife 4, e07836. 10.7554/eLife.07836 PMC4541496 26196144

[acel12651-bib-0027] Sulston JE , Horvitz HR (1988) Methods In The nematode, Caenorhabditis elegans (Wood WB, ed.). Cold Spring Harbor, NY: Cold Spring Harbor Laboratory Press, pp. 587–606.

[acel12651-bib-0028] Van Gilst MR , Hadjivassiliou H , Yamamoto KR (2005) A *Caenorhabditis elegans* nutrient response system partially dependent on nuclear receptor NHR‐49. Proc. Natl Acad. Sci. USA 102, 13496–13501. 16157872 10.1073/pnas.0506234102 PMC1201344

[acel12651-bib-0029] Wang MC , O'Rourke EJ , Ruvkun G (2008) Fat metabolism links germline stem cells and longevity in *C. elegans* . Science 322, 957–960. 18988854 10.1126/science.1162011 PMC2760269

[acel12651-bib-0030] Watts JL , Browse J (2002) Genetic dissection of polyunsaturated fatty acid synthesis in *Caenorhabditis elegans* . Proc. Natl Acad. Sci. USA 99, 5854–5859. 11972048 10.1073/pnas.092064799 PMC122866

[acel12651-bib-0031] Weylandt KH , Serini S , Chen YQ , Su HM , Lim K , Cittadini A , Calviello G (2015) Omega‐3 polyunsaturated fatty acids: the way forward in times of mixed evidence. Biomed. Res. Int. 2015, 143109. 26301240 10.1155/2015/143109 PMC4537707

[acel12651-bib-0032] Yan S , Liang Y , Zhang J , Chen Z , Liu CM (2015) Autoxidated linolenic acid inhibits aflatoxin biosynthesis in *Aspergillus flavus* via oxylipin species. Fungal Genet. Biol. 81, 229–237. 25498164 10.1016/j.fgb.2014.11.005

[acel12651-bib-0033] Zhang M , Wang S , Mao L , Leak RK , Shi Y , Zhang W , Hu X , Sun B , Cao G , Gao Y , Xu Y , Chen J , Zhang F (2014) Omega‐3 fatty acids protect the brain against ischemic injury by activating Nrf2 and upregulating heme oxygenase 1. J. Neurosci. 34, 1903–1915. 24478369 10.1523/JNEUROSCI.4043-13.2014 PMC3905150

