# Supplementary material for: The ω‐3 fatty acid α‐linolenic acid extends Caenorhabditis elegans lifespan via NHR‐49/PPARα and oxidation to oxylipins
Source: Aging Cell. 2017 Aug 3;16(5):1125–35. doi: 10.1111/acel.12651 (PMC5595674; doi:10.1111/acel.12651)
Supplement: Supplementary file 4 — Appendix S1 Additional experimental procedures. [file ACEL-16-1125-s004.docx]

**Additional Experimental Procedures**

***RNAi treatment***

RNAi clones were retrieved from the Ahringer RNAi library and confirmed by sequencing. Clones were grown and then spotted on NGA plates containing 50μg/ml carbenicillin and 1mM IPTG as previously described (Ferguson *et al.* 2010). Due to observed development defects produced by ALA, all worms were added to the plates spotted with RNAi clones, but not containing ALA, as eggs isolated by hypochlorite treatment and grown at 20°C until the worms reached day 1 of adulthood. Hence all worms developed in the absence of ALA supplementation. We were unclear how ALA supplementation would affect the growth and induction of the RNAi expressing bacteria, so the RNAi treatment was only performed during larval development were switched and day 1 adult animals were transferred to fresh plates containing ALA, or the vehicle-only control, that were spotted with OP50-1 bacteria instead of RNAi clones.

***High performance liquid chromatography - electrospray ionization tandem mass spectrometry (HPLC-ESI-MS/MS)***

Oxylipin measurements were conducted with a Thermo Fisher Q Exactive mass spectrometer (ThermoFisher, Waltham, MA) fitted with a PicoChip nanospray source (New Objective, Woburn, MA) and a PicoChip column packed with Waters Atlantis dC18 particles (150 μm x 105 mm; 3 μm particle). The gradient was started at 10 % B for 7 min and then 10 % B to 99 % B over 33 min with the flow rate of 1 μl/min. Mobile phase A is acetonitrile/water (40:60) containing 10 mM ammonium acetate and mobile phase B is acetonitrile/isopropanol (10:90) containing 10 mM ammonium acetate. Data-dependent analyses were conducted using one full MS scan (70,000 resolution) followed by six tandem-MS scans with electrospray negative ion detection. 9(S)-HpOTrE was identified with an accurate mass using a 5-ppm mass tolerance and manual interpretation of the MS/MS fragment patterns. Additionally, a comparison with the retention time of a commercially available standard was used for confirmation.

***Gene Expression Measurement with Nanostring***

For each genotype-RNAi treatment pair, four biological replicates involving RNAi treatment and ALA or control treatment were performed. The worms were washed from the plates in water, and total RNA was isolated from animals by using the Qiagen miRNeasy kit (Valencia, CA). The yield and quality of each RNA sample was evaluated using a Nanodrop spectrophotometer with each sample used for analysis showing A260/A280 ratio ≥1.90 and A260/A230 ratio ≥1.70. Code sets complementary to the indicated genes were synthesized by Nanostring Technologies (Seattle, WA) and used with the Nanostring nCounter system to measure the levels of each transcript in 100 ng aliquots of total RNA. The resulting nCounter data were analyzed using the Nanostring nSolver data analysis software with normalization to the geometric mean of the level of the *cdc-42*, *pmp-3*, and *Y45F10D.4* transcripts in each sample as previously described (Keith *et al.* 2016). The normalized expression data were then exported to Microsoft Excel for further analysis.

***Whole transcriptome sequencing***

RNA-seq studies were performed by treating 10 pairs of biologic replicates with either 5 mM ALA or the vehicle solution without ALA (control) for 18 hours by transferring adult worms to NGA plates containing compounds. Worms were then washed from plates with water and frozen in the RNAlater stabilization solution (ThermoFisher). RNA was extracted by adding Qiazol (Qiagen, Valencia, CA) to the worms suspended in RNAlater, and then homogenizing the sample in a Qiagen Tissuelyser with a 5 mm stainless steel ball. The RNA was then purified using a Qiagen RNeasy kit. The isolated RNA was quantified with a fluorimeter and then assayed for RNA quality using a Caliper GX system (PerkinElmer). Only RNA samples containing more than 1 μg of RNA and having an RNA Integrity Number (RIN) score > 8 were used for further analysis.

The RNA was converted to Illumina sequencing libraries using the Illumina Truseq RNA Sample Preparation Kit (Illumina, San Diego, CA). Using the kit, 1 μg of total RNA is oligo-dT purified using oligo-dT coated magnetic beads, fragmented and then reverse transcribed into cDNA. The cDNA is fragmented, blunt-ended, and ligated to indexed, barcoded adaptors, and then amplified using 15 cycles of PCR. The final library size distribution is validated using capillary electrophoresis and quantified using fluorimetry. The indexed libraries were then normalized, pooled and size selected to 320bp +/- 5% using a Caliper XT instrument. The libraries were sequenced using a 50 basepair paired-end protocol with an Illumina HiSeq 2500 instrument to a goal depth of 10 million reads per library.

The resulting sequencing data was de-multiplexed using the Illumina CASAVA software version 1.8.2 and the resulting FASTQ files were uploaded to the Galaxy server for analysis. The sequences were mapped to the *C. elegans* genome using the HISAT2 program (Kim *et al.* 2015). The mapped reads were then converted into gene counts using the htseq-count program which was run in the “intersection(nonempty)” mode without strandedness and with a minimum alignment quality value of 10 (Anders *et al.* 2015). The gene counts from the pool of control and ALA-treated samples were compared and analyzed for statistical significance using the DESeq2 program using the local fit option and with the outlier fit and replacement options enabled (Love *et al.* 2014). Genes showing a p-value < 0.05 after adjustment for multiple testing using the Benjamini-Hochberg method were considered to be differentially expressed. The differentially expressed genes were then manually annotated using Wormbase, and the online DAVID database to identify biologic themes and determine the magnitude of the fold enrichment (Dennis *et al.* 2003). For DAVID, grouping terms were considered to be meaningful if the provided false-discovery rate was 5% or less.

**References:**

Anders S, Pyl PT, Huber W (2015). HTSeq--a Python framework to work with high-throughput sequencing data. *Bioinformatics*. **31**, 166-169.

Dennis G, Jr., Sherman BT, Hosack DA, Yang J, Gao W, Lane HC, Lempicki RA (2003). DAVID: Database for Annotation, Visualization, and Integrated Discovery. *Genome Biol*. **4**, P3.

Ferguson AA, Springer MG, Fisher AL (2010). skn-1-Dependent and -independent regulation of aip-1 expression following metabolic stress in Caenorhabditis elegans. *Mol Cell Biol*. **30**, 2651-2667.

Keith SA, Maddux SK, Zhong Y, Chinchankar MN, Ferguson AA, Ghazi A, Fisher AL (2016). Graded Proteasome Dysfunction in Caenorhabditis elegans Activates an Adaptive Response Involving the Conserved SKN-1 and ELT-2 Transcription Factors and the Autophagy-Lysosome Pathway. *PLoS Genet*. **12**, e1005823.

Kim D, Langmead B, Salzberg SL (2015). HISAT: a fast spliced aligner with low memory requirements. *Nat Meth*. **12**, 357-360.

Love MI, Huber W, Anders S (2014). Moderated estimation of fold change and dispersion for RNA-seq data with DESeq2. *Genome Biol*. **15**, 550.
